# Investigating the Influence of Biological Sex on the Behavioral and Neural Basis of Face Recognition

**DOI:** 10.1523/ENEURO.0104-17.2017

**Published:** 2017-05-09

**Authors:** K. Suzanne Scherf, Daniel B. Elbich, Natalie V. Motta-Mena

**Affiliations:** 1 Department of Psychology, Pennsylvania State University, University Park, PA 16802; 2 Department of Neuroscience, Pennsylvania State University, University Park, PA 16802

**Keywords:** face processing, fMRI, fusiform gyrus, own-gender bias, sex differences, vision

## Abstract

There is interest in understanding the influence of biological factors, like sex, on the organization of brain function. We investigated the influence of biological sex on the behavioral and neural basis of face recognition in healthy, young adults. In behavior, there were no sex differences on the male Cambridge Face Memory Test (CFMT)+ or the female CFMT+ (that we created) and no own-gender bias (OGB) in either group. We evaluated the functional topography of ventral stream organization by measuring the magnitude and functional neural size of 16 individually defined face-, two object-, and two place-related regions bilaterally. There were no sex differences in any of these measures of neural function in any of the regions of interest (ROIs) or in group level comparisons. These findings reveal that men and women have similar category-selective topographic organization in the ventral visual pathway. Next, in a separate task, we measured activation within the 16 face-processing ROIs specifically during recognition of target male and female faces. There were no sex differences in the magnitude of the neural responses in any face-processing region. Furthermore, there was no OGB in the neural responses of either the male or female participants. Our findings suggest that face recognition behavior, including the OGB, is not inherently sexually dimorphic. Face recognition is an essential skill for navigating human social interactions, which is reflected equally in the behavior and neural architecture of men and women.

## Significance Statement

This research addresses whether there are key differences related to biological sex in the functional organization of the brain. Face processing is one of only a small number of domains in which there is an existing literature suggesting that sex differences in brain function may exist. We provide the most methodologically rigorous test for potential sex differences in face recognition behavior and neural function to date. In so doing, we do not observe such sex differences. We suggest that previous findings could actually reflect group differences in health histories for concussion or emerging psychiatric disorders. Finally, we recommend that research investigating the influence of biological sex become more methodologically rigorous and theory-driven.

## Introduction

There is growing interest in understanding the influence of biological factors, like sex, on the organization of the brain (Cahill, 2014). We have argued that face processing is an ideal system in which to study potential sex differences on behavior and brain function (Scherf et al., 2012). Face perception involves many component processes with varying computational demands (e.g., identity recognition, expression categorization, social attribution), any of which may be influenced by sex. Here, we use a fine-grained approach to investigating sex differences in the behavioral and neural basis of face recognition, the process of identifying individuals, which is critical for navigating and maintaining social relationships.

The existing literature regarding sex differences in face recognition behavior is conflicted. On one hand, empirical findings suggest that women recognize faces more accurately than men (Hall, 1978; Herlitz and Yonker, 2002; Lewin and Herlitz, 2002; Rehnman and Herlitz, 2007). This difference is reportedly a medium-sized effect (Herlitz and Lovén, 2013) and more apparent when women recognize other female faces (Witryol and Kaess,1957; Lewin and Herlitz, 2002; Herlitz and Lovén, 2013), which is refered to as the own-gender bias (OGB). The OGB for males is reported less often (McKelvie et al., 1993; Shapiro and Penrod, 1986; Wright and Sladden, 2003). On the other hand, a meta-analysis concluded that sex differences in face recognition are nonexistent (Shapiro and Penrod, 1986). In sum, there is no consensus on sex differences in face recognition behavior.

Studies investigating sex differences in the neural basis of face processing are sparse and more conflicting. Most of this work has employed electroencephalography and focused on lateralization effects observed during passive viewing of faces. Some (Proverbio et al., 2009), but not all (Allison et al., 1999), studies have reported more right-lateralized responses to faces in men. The fMRI studies are particularly divergent in the pattern of results. For example, Lovén and colleagues reported no sex differences in a whole brain analysis comparing men and women during passive viewing of faces (Lovén et al., 2014). However, in their uncorrected region of interest (ROI) analyses, they reported that both men and women exhibited higher magnitude responses to female versus male faces in the bilateral fusiform and inferior occipital gyri. The authors interpreted these findings to reflect an OGB in women but not men. There is only one fMRI study that evaluated sex differences in neural activation during face recognition (Ino et al., 2010). Using an event-related design with a small number of trials (∼15), the authors reported more activation in the right temporo-parietal junction for women but more activation in the bilateral fusiform gyri and regions outside the face-processing network in men. Neither group exhibited an OGB. In sum, these neuroimaging results fail to converge on a consistent pattern of findings regarding sex differences or an OGB in neural activation, particularly within regions implicated in face recognition.

To address this gap in the literature, we designed a rigorous test of the influence of biological sex on face recognition behavior and its underlying neural architecture. First, we evaluated recognition behavior for male and female faces as well as objects in a large sample of typically developing healthy adults. We created a new long form version of the classic Cambridge Face Memory Test (CFMT+; Duchaine and Nakayama, 2006; Russell et al., 2009) with female faces (F-CFMT+). Second, using fMRI, we investigated the potential influence of sex on the topography of ventral stream organization, and whether there is differential activation (i.e., OGB) for either male or female participants in neural activation explicitly during a face recogniton task. To so do, we scanned each of our participants during two tasks and evaluated the potential influence of sex on three dependent variables related to neural activation (i.e., magnitude, extent, locus) in 16 face-related regions and four control regions using state-of-the-art correction procedures. As a result, this study represents unprecedented rigor in methodological and analytic sensitivity to evaluate the influence of sex on the neural basis of face recognition.

## Materials and Methods

### Participants

Typically developing young adults (*N* = 116, range 18-25 years, 58 females) participated in the behavioral portion of the experiment. Participants were healthy and had no history of neurologic or psychiatric disorders in themselves or their first-degree relatives. Men and women did not differ in age (men: Mean = 19.90 years, SD = 1.75; women: Mean = 19.81 years, SD = 1.69), *t*_(114)_ = 0.27, *p* = 0.79. Individuals who also passed MRI safety screening, had normal or corrected vision, were right handed, and had no history of head injuries or concussions were eligible for the scanning experiment. Given recent evidence that face recognition abilities vary greatly among typically developing adults (Elbich and Scherf, 2017), we also selected participants who did not exhibit extreme deviations in behavioral performance (i.e., within ±1.75 SDs of the grand mean on the M-CFMT+ and F-CFMT+). Our scanning sample included 15 men and 15 women ages 18-25 years (men 20.3 ± 2.3; women 20.6 ± 2.0), who were also not different in age, *t*_(28)_ = 0.43, *p* = 0.67. Note that these inclusion and exclusion criteria were designed to help improve accuracy of estimating any potential sex differences in the experiment by reducing unrelated variability (e.g., due to differences in mental illness symptoms) in our scanned sample that likely exists between men and women in the population at large (Snedecor and Cochran, 1980; Rosnow and Rosenthal, 2008). Also, given our sample size, we followed the most recent recommendations about how to improve statistical power in our analyses (Poldrack et al., 2017).

Written informed consent was obtained using procedures approved by the Internal Review Board of the Pennsylvania State University. Participants were recruited through the Psychology Department undergraduate subject pool and via fliers on campus. Participants were tested in the laboratory on a battery of face and object recognition tasks.

A subset of the participants in the experiment also participated in other neuroimaging experiments that were recently published (Elbich and Scherf, 2017; Elbich et al., unpublished observation). However, none of the analyses reported in this paper have been previously published.

### Behavioral measures

#### Sexual preference questionnaire

This was a two-item questionnaire in which participants provided a self-report assessment of their sexual preference and sexual orientation. The sexual preference question asked about the sex of participants’ preferred sexual partners on a scale of 1-6 (1, women exclusively; 2, women predominantly; 3, both women and men; 4, men predominantly; 5, men exclusively; 6, other). The sexual orientation question asked participants to self-report their orientation using one of four labels, including: 1, heterosexual; 2, homosexual; 3, bisexual; or 4, other. Men reported preferring women exclusively (M = 1.13, SD = 0.51) and being predominantly heterosexual (M = 1.13, SD = 0.52). Women reported preferring men predominantly (M = 4.2, SD = 1.4) and being predominantly heterosexual (M = 1.2, SD = 0.56).

#### Male CFMT (long form; CFMT+)

**Figure 1.**
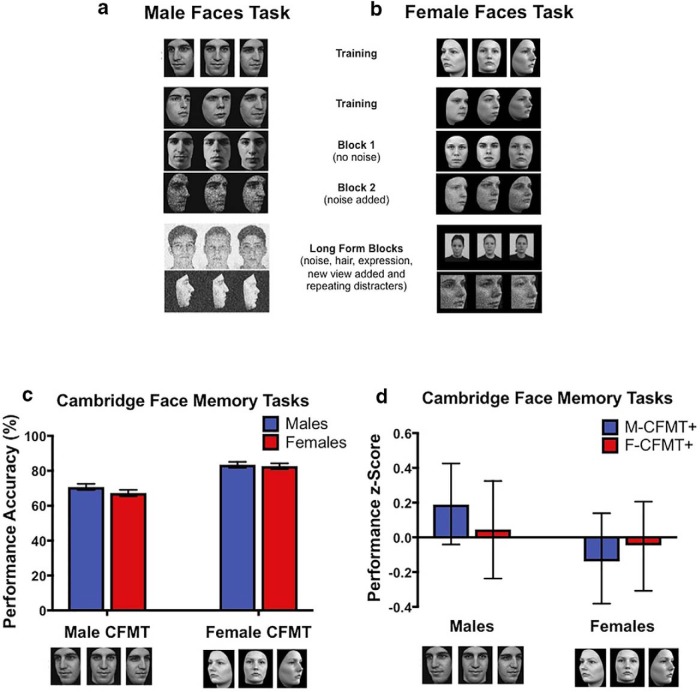
Comparison of performance on the male and female Cambridge Face Memory Task (long form) CFMT+ plotted as a function of sex of participant group. Task outlines of the (***A***) male (figure adapted from Russell et al., 2009) and (***B***) female (created for this experiment) versions of the CFMTs long form (images of female faces are published with permission from the Rafd and KDEF databases and include images AF16NES, AF19NES, and AF29NES). In these tasks, participants view target identities at multiple viewing angles and then must recognize the target faces among distractors with increasing levels of difficulty across blocks, which add noise with changes in lighting and viewpoint (block 2), visual noise (block 3), hair, affect, and repeating distractors (block 4). ***C***, Mean of raw scores for accuracy with 95% inferential confidence intervals (ICIs) for male and female participant groups on each task for the entire sample of 116 participants. Note that the ICIs for male and female participants overlap on the M-CFMT+ and on the F-CFMT+, indicating that there is no sex difference on either task. However, there was a main effect of task, such that both groups performed more accurately on the F-CFMT+ than on the M-CFMT+, which prevented us from computing an unbiased estimate of the OGB and required us to compute standardized scores to do so. ***D***, Standardized accuracy scores with 95% confidence intervals for male and female participants on each task for the entire sample of 116 participants. There was no pattern of OGB for either male or female participants.

The M-CFMT+ is a test of unfamiliar face recognition (Duchaine and Nakayama, 2006; Russell et al., 2009). We used the long form that has previously been used to identify super face-recognizers (Russell et al., 2009). In the task, participants study six target faces with no hair and neutral expressions in each of three viewpoints (Fig. 1*A*). During recognition trials, participants identify target faces in a three-alternative forced choice paradigm under conditions of increasing difficulty. The long form includes an additional set of trials that introduce hair and expressions on the target faces and in which the distractor identities repeat. There are a total of 102 trials.

#### Female CFMT (F-CFMT+)

We created a female version of the CFMT+. This task paralleled all of the parameters of the M-CFMT+, including the editing and presentation of the face images, stimulus timing, and response protocol. As in the M-CFMT+, the photographs were high-resolution images of individual women in the age range between 20-30 years and were selected from the Radboud (Langner et al., 2010) and Karolinska (Lundqvist et al., 1998) faces databases. To make the noise distorted images for blocks 3 and 4 of the task, as in the M-CFMT+, we applied a 30% level of Gaussian noise to the images (Duchaine and Nakayama, 2006). However, given the high resolution of the female face images, we had to apply multiple iterations of this level of noise to degrade the images to a similar level as in blocks 3 and 4 of the M-CFMT+ (Fig. 1*B*).

#### Car Cambridge Memory Test (CCMT)

The CCMT employs the same structure as the CFMT (Dennett et al., 2011), requiring recognition across lighting and view changes, thereby matching the CFMT tasks in general cognitive requirements (e.g., memory, processing speed). Participants study six target cars and subsequently identify the targets in a three alternative forced-choice paradigm under conditions of increasing difficulty. There are 72 trials in this task.

### Neuroimaging protocol

Before scanning, all participants were placed in a mock MR scanner for ∼20 min and practiced lying still. This procedure is highly effective at acclimating participants to the scanner environment and minimizing motion artifact and anxiety (Scherf et al., 2015). During this mock scanning session, participants engaged in a practice version of the scanner recognition task and encoded four exemplars of each of the target male and female identities. An adult male face and an adult female face were presented side-by-side and labeled as “John” and “Jane.” Participants were given 10 seconds to encode the faces. Following this, participants engaged in four practice blocks of the task (two male and two female) using novel exemplar and distractor faces. The task involved looking at blocks of 12 sequentially presented faces and identifying the two target identities among 10 distractor faces. No stimuli from the practice task were used in the scanner task (see below).

Participants were scanned using a Siemens 3T Trio MRI with a 12-channel phase array head coil at the Social, Life, and Engineering Imaging Center (SLEIC) at Penn State University. During the scanning session, visual stimuli were displayed on a rear-projection screen located inside the MR scanner.

#### Visual stimulation task

A visual stimulation task was created to activate face-, object-, and place-selective regions in individual participants (Elbich and Scherf, 2017). Tasks with dynamic stimuli are better at eliciting face-related activation throughout multiple nodes of the distributed face-processing network (Fox et al., 2009; Pitcher et al., 2011). Notably, the magnitude of activation in response to dynamic and static images does not differ in the right fusiform face area (FFA) (Pitcher et al., 2011). Given that our goal was to investigate the potential influence of biological sex in as many nodes of the face-processing network as we could define, we employed a dynamic task that would provide a better chance of identifying activation throughout this distributed network.

The task was a single run and included blocks of silent, fluid concatenations of short movie clips from four conditions: unfamiliar faces, famous faces, common objects, and navigational scenes. The short (3-5 s) video clips in the stimulus blocks were taken from YouTube and edited together using iMovie. The movie clips of faces were intensely affective (e.g., a person yelling or crying) to elicit activation throughout the network of core and extended face-processing regions (Gobbini and Haxby, 2007). Movies of objects included moving mechanical toys and devices (e.g., dominos falling). Navigational clips included panoramic views of nature scenes (e.g., oceans, open plains). The task was organized into 24 16-s stimulus blocks (six per condition). The order of the stimulus blocks was randomized for each participant. Fixation blocks (6 s) were interleaved between task blocks. The task began and ended with a 12-s fixation block. Following the first fixation block, there was a 12-s block of patterns. The task was 9 min and 24 s.

#### Identity recognition task

This task was designed to roughly mirror the computational demands of the fourth block of the CFMTs. Participants had to recognize novel exemplars (new expressions, new hair styles, new lighting, new viewpoint) of the target face identities that they encoded during the mock scanning session among a series of distractor faces. The task was a blocked paradigm that included a total of 20 12-s blocks (10/sex) interleaved with 6-s fixation blocks. Within each block, stimuli were presented for 800 ms followed by a 200-ms fixation. Twelve unique images were presented sequentially in each block, with two of the images being novel exemplars of the target faces and while the other 10 served as novel identities/distractors. Participants were required to press a button each time they identified the target identity male or female face. The task began with a 12-s block of fixation that was followed by a 12-s block of visual patterns and 6-s fixation block. The task concluded with a 12-s block of fixation. The task was 6 min and 36 s and was executed in a single run.

#### MRI data acquisition

Functional EPI images were acquired in 34 slices (3 mm thick; TR = 2000 ms; TE = 25; flip angle = 80°, FOV = 210 × 210, 3 mm isotropic voxels). The functional images were aligned ∼30° in the rostral direction from the AC-PC line (e.g., approximately perpendicular to the hippocampus), which minimizes noise from the eye orbits and nasal sinuses and maximizes signal in the medial temporal lobes (Whalen et al., 2008). This protocol allowed for full coverage of the ventral visual pathway in the temporal lobe as well as of the frontal and occipital lobes. For participants with larger head size, the superior parietal lobe was not completely covered. Anatomic images were collected using a 3D-MPRAGE with 176 straight sagittal slices (1 mm, T1-weighted, TR = 1700; TE = 1.78; flip angle = 9°; FOV = 256).

### Data analysis

#### Behavioral data

Accuracy was recorded for all behavioral tasks. Before the analyses, we examined the behavioral data for outliers and violations of normality separately for each behavioral task within each sex, and group differences in variance. Group differences were evaluated using a repeated-measures ANOVA including the within-subjects factor of stimulus sex (female, male) and the between-subjects factor of participant sex (female, male). Initial analyses revealed consistent task, but not sex, differences across both male and female participants between the three recognition memory tasks (i.e., main effects and no interactions). As a result, we *z*-transformed the raw accuracy scores for each task so that scores on the two CFMT+ tasks could be compared directly for the presence of differential OGB effects. The *z*-scores were then submitted to the repeated-measures ANOVAs to investigate the potential sex of participant by sex of stimulus interactions. As in previous studies, we also used the *z*-transformed CCMT scores as a covariate in the repeated-measures ANOVA investigating group differences on the CFMT scores because this provides a strategy for removing any potential sex differences related to general memory abilities related to the task design (Dennett et al., 2011) in the analysis of potential sex differences in face recognition. Planned comparisons contrasting group performance on each task were investigated using two-tailed independent samples *t*-tests. We also employed a method for calculating inferential confidence intervals (ICIs) that was developed by Tryon and colleagues (Tryon, 2001; Tryon and Lewis, 2008) and has been used to evaluate group differences in recent patient work (Avidan et al., 2014). It addresses some issues with traditional null hypothesis testing methods and enables one to infer statistical difference (or lack thereof) between two groups. For each behavioral test, we compared the males and females in accuracy to determine if they were statistically different using this method with the 95% ICIs (α = 0.05). Nonoverlapping ICIs indicate a statistical difference.

#### Neuroimaging data

Functional volumes were preprocessed, including 3D motion correction, linear trend removal, slice scan time correction, and filtering out low frequencies (three cycles) using BrainVoyager QX version 2.3 (RRID:SCR_013057). Head motion within both functional runs was <3 mm (1 voxel) in all six directions in all volumes for all subjects. Separate independent samples *t*-tests on each of the six motion dimensions in both tasks revealed no group differences in movement (all *p* > 0.05). Thus, any group differences in the functional profile of the ventral visual pathway cannot be explained by motion differences between the groups.

For each participant, the time series images for each brain volume for each participant were analyzed for stimulus category and/or experimental condition differences in a fixed-factor general linear model (GLM) for each task. Each category/condition was defined as a separate predictor with a box-car function adjusted for the delay in hemodynamic response. The time series images were then spatially normalized into Talairach space. The functional images were not spatially smoothed (Weiner and Grill-Spector, 2013).

In both the group and individual level analyses of the visual stimulation task, the time series images for each brain volume for each individual participant were used to define category selectivity. As in previous studies using this kind of task, we adopted a conservative definition of category selectivity contrasting the averaged BOLD response amplitude (across blocks within a category) for each category to that of all the others (Hasson et al., 2004; Avidan et al., 2005; Scherf et al., 2007; Humphreys et al., 2008; Golarai et al., 2010; Elbich and Scherf, 2017). For example, face-selective activation was defined by the contrast [*Famous + Unfamiliar Faces*] > [*Objects + Navigation*]. Object-related activation was defined by the weighted contrast 3*[*Objects*] > [*Famous Faces + Unfamiliar Faces + Navigation*]. Finally, place-related activation was defined by the weighted contrast 3*[*Navigation*] > [*Famous Faces + Unfamiliar Faces + Objects*].

#### Evaluating group differences in category selectivity using the visual stimulation task

We used a two-pronged approach to evaluate potential sex differences in the topography of category-selective activation of the ventral visual pathway and the extended face-processing regions. First, we compared the groups in whole-brain level contrasts for each kind of category-selective activation (faces, objects, places). Second, we identified category-selective activation in individually-defined a priori ROIs and compared multiple dependent measures quantifying these ROIs, including the magnitude of activation, functional size of the ROI, and locus of each region.

#### Whole-brain group level comparisons of category-selective activation

Category selectivity was initially evaluated separately in each group by submitting the individual subject time series images to a whole brain voxel-wise random effects GLM in which the category was a fixed factor and participant was a random factor. To examine potential sex differences in face-, object, and place-related activation, the fMRI data from the two groups were compared directly in a whole-brain voxel-wise mixed-model ANOVA including group and visual category as fixed factors and subject as a random factor. Group differences for activation related to each visual category were examined using the following balanced interactions, whereby *Faces* includes the two blocks of famous and unfamiliar faces:

[Female (*Faces*) > (*Objects+Navigation)]* ***>*** [Male (*Faces*) > (*Objects+Navigation)*]

[Female 3*(*Objects*) > (Faces*+Navigation)*] **>** [Male 3*(*Objects*) > (*Faces+Navigation)*]

[Female 3*(*Navigation*) > (*Faces+Objects*)] **>** [Male 3*(*Navigation*) > (*Faces+Objects*)].

The group maps were corrected for multiple comparisons using the false discovery rate (FDR) procedure with *q* < 0.05 (Genovese et al., 2002).

#### Group level comparisons of category selective activation within individually defined ROIs

To evaluate whether there were sex differences in the size or magnitude of activation within a priori defined ROIs, the functional profile of category-selective activation was determined in individually defined ROIs for each participant in each group. These ROIs were extracted from separate contrast maps (face-, place-, object-selective ROIs as defined above) in each participant that were corrected for multiple comparisons using the FDR procedure of *q* < 0.001 (Genovese et al., 2002).

ROIs of both the core (FFA, OFA, pSTS) and extended (amygdala, vmPFC, PCC, anterior temporal lobe) face processing regions were defined for each participant in each hemisphere separately using the face contrast (see above). We also defined multiple face ROIs within the fusiform gyrus. The nomenclature for the multiple patches in the fusiform gyrus varies (Weiner et al., 2014; Engell and McCarthy, 2013). The cluster of contiguous voxels nearest the classically defined FFA (i.e., Talairach coordinates right: 40, -41, -21, left: -38, -44, -19) in the middle portion of the gyrus was identified as the pFus-faces/FFA1 (Weiner and Grill-Spector, 2010). Functional activation between this ROI and the anterior tip of the mid fusiform sulcus, was called the mFus-faces/FFA2 (Weiner and Grill-Spector, 2010), and activation posterior to FFA1 within the fusiform gyrus and rostral to the posterior transverse collateral sulcus was called the posterior FG/IOG. The region we call posterior FG/IOG is sometimes identified by other groups as the IOG/OFA (Weiner et al., 2014); however, there is controversy about the locus of the OFA and whether there are multiple OFAs in the inferior occipital gyrus (Pitcher et al., 2011). Consequently, we defined the OFA as the set of contiguous voxels on the lateral surface of the occipital lobe closest to our previously defined adult group level coordinates (right: 50, -66, -4, left: -47, -70, 6; Scherf et al., 2007). The pSTS was defined as the set of contiguous voxels within the horizontal posterior segment of the superior temporal sulcus (right: 53, -50, 11; left: -53, -52, 14) that did not extend into the ascending posterior segment of the STS. The most anterior boundary of the pSTS was where the ascending segment of the IPS intersected the lateral fissure. The anterior temporal lobe ROI was defined as the cluster of voxels nearest the coordinates reported previously in studies of individual face recognition (right: 35, -3, -25; left: -26, -6, -27; Mur et al., 2010), which is at the most anterior tip of the collateral sulcus and fusiform gyrus, between the occipitotemporal sulcus and the parahippocampal gyrus. The PCC was defined as the cluster of voxels in the posterior cingulate gyrus above the splenium of the corpus callosum near the coordinates reported in previous studies of face processing (0, -51, 23; Schiller et al., 2009). The vmPFC was defined as the cluster of voxels in the medial portion of the superior frontal gyrus ventral to the cingulate gyrus near coordinates reported in previous studies of social components of face processing (0, 48, -8; Schiller et al., 2009). The amygdala was defined as the cluster of face-selective voxels within the gray matter structure. Any active voxels that extended beyond the structure out to the surrounding white matter, horn of the lateral ventricle, or hippocampus were excluded.

Object-related activation was identified in the lateral occipital complex (LOC). The object-related ROI included the set of contiguous object-selective voxels on the lateral surface of the occipital lobe in the middle occipital gyrus that were nonoverlapping with the voxels identified in the OFA ROI. Navigation-related activation was identified in the parahippocampal place area (PPA) and included the contiguous navigation-selective voxels in the parahippocampal gyrus (as determined by the maximal *x*-, *y*-, and *z*-coordinates of BAs 34, 35, and 36 in the Talairach atlas). Critically, these contrasts identify nonoverlapping sets of voxels in all participants, indicating that they identify the most selective of voxels for each visual category.

Note that our voxel selection criteria (i.e., ROI definitions) were defined a priori based on an existing model of face processing (Gobbini and Haxby, 2007), corrected at the whole-brain level for false positive activations (FDR *q* < 0.001), and completely independent of group-related contrasts. As a result, the subsequent analyses comparing the dependent measures from the ROIs between groups are orthogonal to the voxel-selection process (Poldrack and Mumford, 2009; Nichols and Poline, 2009; Vul et al., 2009). This analysis approach is consistent with that used in many other group comparison neuroimaging experiments (Golarai et al., 2007, 2010; Scherf et al., 2007, 2015; Avidan et al., 2014; Elbich and Scherf, 2017).

Table 1 illustrates the total number of participants for whom each ROI was definable together with the average coordinates for the centroid of each ROI. The ROIs were quantified in terms of the total number of significantly active voxels. As in previous research, a score of 0 was entered for each ROI in which a participant did not exhibit any significantly active voxels (Golarai et al., 2007, 2015; Scherf et al., 2007, 2010, 2015). To compute the magnitude of category selectivity within each ROI in each hemisphere, separate ROI-based GLMs were conducted for each participant who exhibited identifiable category-selective activation in each ROI. This generated β weights for each condition (i.e., faces, objects, navigation) for each participant. For each person, in each ROI, we computed selectivity (see contrasts above) for each visual category using the β weights. Participants with no identifiable voxels in an ROI were excluded from the analyses of selectivity, given that no ROI-based GLM could be computed.

**Table 1.** Summary of definable ROIs from the visual stimulation experiment

			Mean region coordinatesmale participants					Mean region coordinatesfemale participants			
Category	ROI	*N*	X	Y	Z	Size (SD)	*N*	X	Y	Z	Size (SD)
**Faces**											
Coreregions	R fusiformgyrus										
	*m-Fus (FFA2)*	7	38 (6)	−26 (6)	−19 (5)	305 (516)	9	37 (3)	−41 (16)	−18 (3)	306.6 (529)
	*p-Fus (FFA1)*	15	37 (4)	−45 (6)	−20 (3)	1384.2 (908)	15	38 (4)	−45 (7)	−20 (4)	1959.7 (1424)
	*pFG (IOG)*	15	37 (5)	−72 (7)	−14 (10)	1008.2 (565)	14	36 (5)	−75 (6)	−17 (5)	1231.3 (1279)
	L Fusiform Gyrus										
	*m-Fus (FFA2)*	5	−38 (6)	−25 (8)	−21 (4)	118.8 (231)	9	−41 (5)	−36 (16)	−20 (4)	320 (424)
	*p-Fus (FFA1)*	15	−40 (3)	−45 (5)	−20 (3)	1074 (626)	14	−41 (4)	−45 (7)	−21 (5)	1375.5 (979)
	*pFG (IOG)*	14	−40 (5)	−69 (6)	−19 (3)	867.3 (1039)	15	−40 (4)	−75 (6)	−17 (6)	1044.7 (1024)
	R OFA	14	46 (3)	−62 (6)	4 (5)	1806.6 (1357)	14	44 (6)	−64 (6)	4 (5)	1909.33 (1648)
	L OFA	13	−50 (5)	−65 (7)	6 (5)	1166.4 (1317)	14	−49 (7)	−66 (7)	6 (5)	1181.5 (1029)
	R STS	15	50 (4)	−41 (6)	8 (5)	2585.1 (1795)	15	50 (5)	−40 (6)	6 (5)	2814.7 (1925)
	L STS	14	−56 (6)	−43 (6)	7 (5)	1511.2 (1486)	14	−55 (7)	−44 (4)	5 (6)	1048.6 (918)
Extended regions	vmPFC	13	2 (3)	47 (8)	−8 (6)	594.1 (650)	11	3 (4)	50 (6)	−7 (5)	806.3 (1051)
	PCC	13	1 (3)	−51 (5)	25 (7)	890 (1287)	10	3 (3)	−53 (5)	22 (7)	567.3 (831)
	R Amyg	12	18 (4)	−6 (2)	−12 (2)	128 (152)	10	17 (3)	−7 (2)	−11 (3)	206.5 (299)
	L Amyg	12	−18 (3)	−6 (5)	−12 (2)	85 (143)	8	−19 (2)	−7 (2)	−11 (2)	170.3 (296)
	R ATP	13	36 (4)	1 (7)	−31 (5)	154 (220)	13	32 (9)	3 (9)	−30 (5)	183.8 (320)
	L ATP	9	−37 (5)	−2 (6)	−27 (5)	72.5 (116)	7	−37 (4)	−3 (5)	−28 (6)	70.4 (183)
**Places**	R PPA	15	30 (10)	−44 (9)	−10 (3)	439 (433)	12	25 (4)	−41 (8)	−13 (5)	338.7 (464)
	L PPA	13	−27 (4)	−45 (8)	−11 (4)	333.6 (333)	10	−29 (4)	−43 (8)	−12 (5)	170.5 (341)
**Objects**	R LOC	14	46 (4)	−62 (6)	−9 (7)	2186.5 (1746)	14	46 (5)	−58 (6)	−11 (4)	1308.3 (1925)
	L LOC	15	−47 (5)	−65 (5)	−9 (3)	3215.5 (1686)	14	−47 (3)	−64 (5)	−8 (6)	2740.9 (529)

*p < 0.05, **p < 0.01, ***p < 0.005, ****p < 0.001.

Given the previous reports of differences in the profile of lateralization in activation across men and women, we evaluated potential group differences in neural activation elicited during the visual stimulation task by submitting the magnitude contrast scores for each pair of bilateral ROIs to separate repeated-measures ANOVAs with hemisphere as the within-subjects factor and group as the between-subjects factor. We analyzed the potential group differences in the extent of activation for each pair of bilateral ROIs as well. Importantly, in these analyses, because hemisphere is a within-subjects factor, only participants who have defined ROIs in both hemispheres contributed to each analysis. As a result, most of these analyses do not have the full set of participants. Therefore, as a set of follow-up analyses, we also performed a series of independent samples *t*-tests within each ROI investigating group differences on each measure so that we could leverage the power of the full sample to understand whether biological sex influenced the measure of neural magnitude or extent of activation within each of these regions. When the variance was unequal for a particular measure within an ROI, we report the *t*-test with equal variance not assumed. In each analyses, we maintained a familywise error correction rate of 0.05/12 = 0.004, which takes into consideration the two dependent variables (magnitude, extent of activation), two hemispheres, three types of category-specific activation (face, object, place).

#### Evaluating group differences in neural activation during the identity recognition task

To evaluate potential group differences in the neural responses generated during an active face recognition task, the analyses began at the individual level. For each individual participant, we submitted the time series data from the Identity Recognition task to compute separate ROI-based GLMs with the factor of stimulus sex for each of the independently defined face-related ROIs (i.e., bilateral pFG/IOG, p-Fus/FFA1, m-Fus/FFA2, OFA, pSTS, amygdala, PCC, vmPFC). From these a priori selected independent voxels, we extracted the mean β weights for male and female faces separately. We submitted these extracted parameter estimates from each pair of bilateral ROIs to separate repeated-measures ANOVAs with hemisphere (2) and stimulus sex (2) as the within-subjects factors and group as the between-subjects factor. As in the analyses of the visual stimulation ROI data, because hemisphere is a within-subjects factor, only participants who have defined ROIs in both hemispheres contributed to each analysis. As a result, most of these analyses do not have the full set of participants. To that end, as a set of follow-up analyses, we computed an OGB score for each participant from the parameter estimates. For male participants this was computed as [male faces-female faces] and for female participants it was computed as [female faces – male faces]. For each ROI, we submitted these bias scores to separate independent samples *t*-tests to investigate potential group differences. This approach allowed us to leverage the power of the full sample to investigate potential sex differences in the magnitude of the neural responses during face recognition and whether they were biased in an own-gendered way. When the variance was unequal, we report the *t*-test with equal variance not assumed. In these analyses, we maintained a familywise error correction rate of 0.05/12 = 0.004, which takes into consideration the stimulus sex (male, female), two hemispheres, three types of category-specific activation (face, object, place).

## Results

### Reliability of behavioral tasks

To evaluate the reliability of performance on the F-CFMT+, we calculated the correlation between each section as a measure of task consistency across participants as in Duchaine and Nakayama (2006). In both tasks, all participants performed near ceiling in the instruction block, preventing us from evaluating variability in performance in this block with other blocks. However, for the F-CFMT+, performance on the block of novel images (block 2) was highly consistent with performance on the block with noise (block 3; *r* = 0.58, *p* < 0.0001), and the block with affect, noise, hair, new viewing angles, increased noise and new distractors (block 4; *r* = 0.18, *p* = 0.05). Finally, performance on blocks 3 and 4 was also highly correlated for the F-CFMT+ (*r* = 0.47, *p* < 0.0001). These results mirrored performance accuracy reported in the original paper describing the M-CFMT short form (Duchaine and Nakayama, 2006) as well as performance in our own sample of participants on the M-CFMT+. Specifically, in the M-CFMT+ performance on block 2 was highly consistent with performance on block 3 (*r* = 0.71, *p* < 0.0001), and block 4 (*r* = 0.52, *p* < 0.0001). Additionally, performance on blocks 3 and 4 was also highly correlated for the M-CFMT+ (*r* = 0.53, *p* < 0.0001). Finally, performance across both tasks was highly correlated for both men (*r* = 0.71, *p* < 0.001) and women (*r* = 0.46, *p* < 0.001).

### Biological sex as a factor influencing face recognition behavior

First, we tested potential sex differences in face and object recognition abilities for the larger sample of 116 participants by submitting the raw accuracy scores to a repeated-measures ANOVA. Importantly, there was no main effect of group, *F*_(114)_ = 1.89, *p* = 0.17, and no group × stimulus sex interaction, *F*_(114)_ = 2.90, *p* = 0.09. The 95% ICIs for the males (69.1-72.5) and the females (65.5-69.1) overlapped for performance on the M-CFMT+ as did the ICIs for the males (81.8-85.1) and females (81.0-84.3) on the F-CFMT+ indicating that there were no statistical differences between groups on either task.

However, there was a main effect of task, *F*_(114)_ = 332.39, *p* < 0.001, indicating that both groups were more accurate when recognizing female (M = 83.1, SD = 8.8) compared with male (M = 69.0, SD = 9.8) faces (Fig. 1*C*). This superior performance by both groups on F-CFMT+ in the raw scores presents like a disproportionate female OGB (female > male faces bias), but males have a female face bias in the raw scores as well. What this makes clear is that if the two tasks are not matched in difficulty, one cannot acquire an unbiased assessment of the OGB for each group, which is reflected in a group × sex of stimulus interaction. As a result, we *z*-transformed the scores from all three tasks so that we could compare them in the same distribution. This allowed us to measure an unbiased assessment of the OGB and the potential sex difference in the presence or magnitude of this effect. In the repeated-measures ANOVA using the *z*-transformed scores from the two CFMT+ tasks and the *z*-transformed CCMT scores as a covariate in the analysis, there was no main effect of task, *F*_(113)_ = 0.10, *p* = 0.76. This demonstrates that the distributions of the standardized scores are now comparable so that the interaction between sex of participant and sex of stimulus can be evaluated. Consistent with the previous analysis, there was no main effect of group, *F*_(113)_ = 0.70, *p* = 0.41. The planned comparisons revealed that men and women did not differ in accuracy for recognition of female faces, *t*_(114)_ = 0.49, *p* = 0.63, or male faces, *t*_(114)_ = 1.91, *p* = 0.06 (Fig. 1*D*). Critically, there was no sex of participant × sex of stimulus interaction, *F*_(113)_ = 2.15, *p* = 0.15, indicating no OGB for male or female participants (Fig. 1*D*). This was confirmed by computing paired samples *t*-tests for each group separately to evaluate superior performance on the sex-specific face for each group. Neither the female, *t*_(57)_ = 0.72, *p* = 0.48, nor male, *t*_(57)_ = 1.55, *p* = 0.13, participants exhibited more accurate recognition of sex-specific faces, and the 95% confidence interval for this difference in accuracy overlapped across groups and 0 [males: (−0.04, 0.32); females: (−0.33, 0.17)] reflecting that neither group had superior performance on either task. In contrast, there was a sex difference for object recognition on the CCMT, *F*_(1,112)_ = 10.17, *p* = 0.002, η^2^ = 0.08, revealing that males (M = 77.3, SD = 12.7) were more accurate for recognizing cars compared with females (M = 69.60, SD = 11.40), even after controlling for the task demands by using the M-CFMT+ and F-CFMT+ scores as covariates.

The behavioral results among the subset of 30 individuals who participated in both the behavioral and neuroimaging experiments were similar to the larger sample. When evaluating face recognition performance while controlling for the task demands as assessed by the CCMT, there were no main effects of task, *F*_(1,27)_ = 0.00, *p* = 0.95, or group, *F*_(1,27)_ = 0.76, *p* = 0.39. The planned comparisons between male and female participants in the neuroimaging sample revealed that performance did not differ in accuracy to recognize either female, *t*_(28)_ = 0.50, *p* = 0.62, or male, *t*_(28)_ = 1.47, *p* = 0.15, faces. Also, as in the larger sample, there was no sex of participant × sex of stimulus interaction, *F*_(1,27)_ = 0.79, *p* = 0.38. The planned contrasts revealed that neither the female, *t*_(14)_ = 0.62, *p* = 0.54, nor male, *t*_(14)_ = 0.59, *p* = 0.57, participants exhibited more accurate recognition of sex-specific faces, and the 95% confidence interval for this difference in accuracy overlapped across groups and 0 [males: (−0.29, 0.51); females: (−0.45, 0.26)] reflecting that neither group had superior performance for same-sex faces. Finally, in contrast to the larger sample, there was no main effect of group during object recognition on the CCMT, *F*_(1,26)_ = 0.87, *p* = 0.36. In sum, there were no sex differences in recognition accuracy for male or female faces or cars in the subset of neuroimaging participants. Furthermore, there was no pattern of OGB for either male or female participants in their recognition accuracy for faces in both the full and scanned samples.

### Biological sex as a factor influencing the topography of the ventral visual pathway

**Figure 2.**
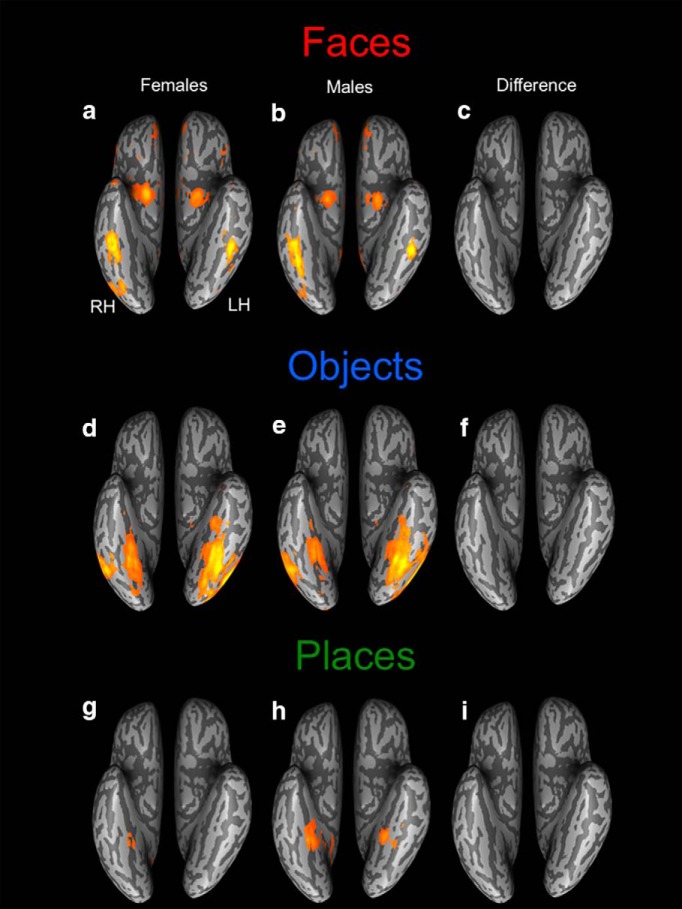
Comparison of category-specific topographic functional organization in the ventral visual pathway between female and male participants. Contrast maps for each visual category from the group-level random effects GLM (FDR *q* < 0.05) for female (***A***, ***D***, ***G***) and male (***B***, ***E***, ***H***) participants. Face-related activation was defined as [*Famous + Unfamiliar Faces*] > [*Objects + Navigation*]. Object-related activation was defined by the weighted contrast 3*[*Objects*] > [*Famous Faces + Unfamiliar Faces* + *Navigation*]. Place-related activation was defined by the weighted contrast 3*[*Navigation*] > [*Famous Faces + Unfamiliar Faces + Objects*]. Direct contrasts between these maps reveal that there are no sex differences in the topographic organization of face-selective (***C***), object-selective (***F***), or place-selective (***I***) activation (all contrasts corrected at whole-brain FDR *q* < 0.05).

The whole-brain analyses of category selective activation for each group from the visual stimulation task are presented in Figure 2. Women (Fig. 2*A*) and men (Fig. 2*B*) both activated face-processing regions bilaterally in the core and extended face-processing network, including the OFA, FFA, pSTS, amygdala, and vmPFC. Critically, there were no sex differences when the groups were compared directly in the whole-brain ANOVA (Fig. 2*C*). When observing heterogeneous common objects, both women (Fig. 2*D*) and men (Fig. 2*E*) exhibited large areas of activation in ventral temporal cortex, including the right and left LOC and the posterior fusiform gyri. As with face processing, there were no sex differences when the groups were compared in the whole-brain ANOVA (Fig. 2*F*) during object viewing. Finally, when looking at navigational scenes, women (Fig. 2*G*) and men (Fig. 2*H*) both activated the bilateral parahippocampal gyri and there were no sex differences resulting from the whole-brain ANOVA (Fig. 2*I*).

Next, we evaluated potential sex differences in the size and magnitude of the individually defined ROIs from the category-selective activation derived from the visual stimulation task. The number of definable regions in each participant, including the location and size of each of the regions, is listed in Table 1. To evaluate any differences in the number of “definable” regions for each group, we performed a χ^2^ test on the number of males and females who had identifiable ROIs for each region. There were no sex differences in the number of identifiable ROIs for any region (all *p* > 0.05; Table 2).

**Table 2.** Comparing the number of male and female participants who had definable functional regions in the visual stimulation experiment

Category	ROI	χ^2^	df	*p*
**Faces**				
Core regions	R fusiform gyrus			
	*m-Fus (FFA2)*	16.29	16	n.s.
	*p-Fus (FFA1)*	30.00	29	n.s.
	*pFG (IOG)*	30.00	29	n.s.
	L fusiform gyrus			
	*m-Fus (FFA2)*	17.08	17	n.s.
	*p-Fus (FFA1)*	30.00	29	n.s.
	*pFG (IOG)*	30.00	29	n.s.
	R OFA	28.00	28	n.s.
	L OFA	27.33	27	n.s.
	R STS	30.00	29	n.s.
	L STS	28.00	28	n.s.
Extended regions	vmPFC			
	PCC	24.67	24	n.s.
	R Amyg	24.29	23	n.s.
	L Amyg	22.50	22	n.s.
	R ATL	21.60	19	n.s.
	L ATL	24.00	24	n.s.
**Places**	R PPA	14.29	15	n.s.
	L PPA	30.00	26	n.s.
**Objects**	R LOC	24.29	22	n.s.
	L LOC	28.00	28	n.s.

**Figure 3.**
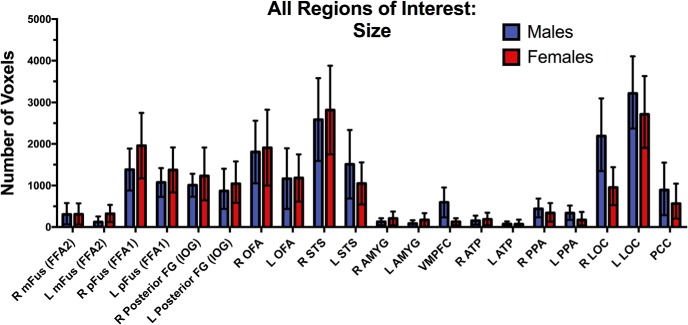
Comparing face-related activation as defined by functional size throughout the face-processing network in a priori ROIs. The ROIs were defined using the visual stimulation task and were thresholded for each individual participant using the FDR procedure (*q* < 0.001). Mean number of significant contiguous voxels in each ROI with 95% confidence intervals plotted as a function of participant sex. There were no sex differences in the functional size of any ROI.

**Figure 4.**
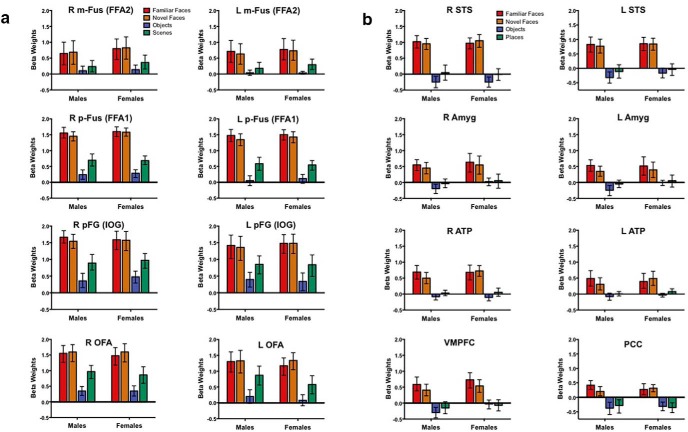
***A***, ***B***, Comparing face-related activation as defined by magnitude throughout the face-processing network in individually defined face-selective ROIs. The ROIs in the core and extended (***A***, ***B***) face-processing network were defined using the visual stimulation task and were thresholded for each individual participant using the FDR procedure (*q* < 0.001). Each graph represents the mean parameter estimates (e.g., β weight) from the ROI-based GLM with 95% confidence intervals for male and female participants. There were no sex differences in the magnitude of the response to faces in any ROI.

Given that the number of definable regions was comparable in men and women, we evaluated whether the magnitude and extent of activation was related to biological sex and/or hemisphere separately in pairs of bilateral individually defined ROIs. There were no main effects of group or group × hemisphere interactions for the size of face-related activation in the face-related ROIs (*p* > 0.004), object-related activation in LOC (*p* > 0.004), or place-related activation in PPA (*p* > 0.05; Table 3; Fig. 3). However, there was a main effect of hemisphere such that face-related activation was larger in extent in the right than left hemisphere for both men and women in the pSTS (*p* < 0.004). Also, there was a main effect of hemisphere in the LOC such that both men and women exhibited larger extent of object-related activation in the left compared with right hemisphere (*p* < 0.004). Similarly, there were no main effects of group, hemisphere, or group hemisphere interactions for the magnitude of face-related activation in the face-related ROIs (*p* > 0.004), object-related activation in LOC (*p* > 0.004), or place-related activation in PPA (*p* > 0.004; Table 3; Fig. 4).

**Table 3.** Summary of results from repeated-measures ANOVAs on the magnitude and size of category-selective activation during the visual stimulation experiment in each pair of bilateral ROIs

	Size				Magnitude		
Category		df	*F*	*p*	df	*F*	*p*
Face ROIs							
Core regions							
p-Fus (FFA2)	*Group*	1, 28	0.576	0.454	1, 12	0.733	0.409
	*Hem*	1, 28	0.917	0.346	1, 12	0.523	0.483
	*Group* × *Hem*	1, 28	1.224	0.278	1, 12	0.186	0.674
m-Fus (FFA1)	*Group*	1, 28	1.638	0.211	1, 28	0.763	0.390
	*Hem*	1, 28	8.829	0.006	1, 28	1.438	0.241
	*Group* × *Hem*	1, 28	0.829	0.370	1, 28	0.165	0.688
pFG (IOG)	*Group*	1, 28	0.436	0.515	1, 25	0.652	0.427
	*Hem*	1, 28	0.609	0.442	1, 25	3.354	0.079
	*Group* × *Hem*	1, 28	0.012	0.914	1, 25	0.928	0.345
OFA	*Group*	1, 28	0.019	0.890	1, 23	1.630	0.214
	*Hem*	1, 28	7.025	0.013	1, 23	2.410	0.134
	*Group* × *Hem*	1, 28	0.029	0.866	1, 23	1.240	0.277
STS	*Group*	1, 28	0.058	0.812	1, 26	0.002	0.969
	***Hem***	**1, 28**	**20.791**	**0.000**	1, 26	2.593	0.119
	*Group* × *Hem*	1, 28	1.235	0.276	1, 26	1.505	0.231
Extended regions							
Amygdala	*Group*	1, 28	1.116	0.300	1, 17	2.446	0.136
	*Hem*	1, 28	1.175	0.288	1, 17	4.085	0.059
	*Group* × *Hem*	1, 28	0.009	0.926	1, 17	0.304	0.589
ATL	*Group*	1, 28	0.053	0.820	1, 13	0.132	0.722
	*Hem*	1, 28	3.200	0.084	1, 13	1.887	0.193
	*Group* × *Hem*	1, 28	0.086	0.772	1, 13	1.599	0.228
**Place ROIs**							
PPA	*Group*	1, 28	1.095	0.304	1, 21	0.016	0.901
	*Hem*	1, 28	3.578	0.069	1, 21	0.005	0.947
	*Group* × *Hem*	1, 28	0.183	0.672	1, 21	0.213	0.649
**Object ROIs**							
LOC	*Group*	1, 28	2.788	0.106	1, 26	1.195	0.284
	***Hem***	**1, 28**	**27.227**	**0.000**	1, 26	8.917	0.006
	*Group* × *Hem*	1, 28	1.864	0.183	1, 26	0.719	0.404

Face-selective activation defined for face ROIs, object-selective activation defined for object ROIs, and place-selective activation defined for place ROIs. Bolded *p* values surpassed the FWE correction of *p* < 0.004.

**Figure 5.**
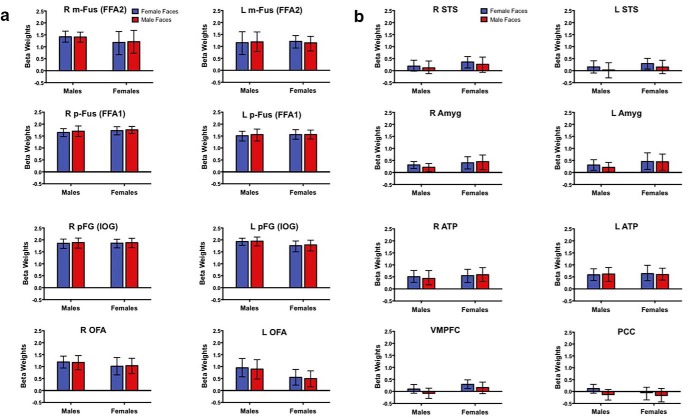
***A***, ***B***, Comparing face-related activation during recognition of female and male faces. Neural activation acquired during recognition of female and male faces in scanner face recognition task plotted as a function of participant sex for each ROI in the core (***A***) and extended (***B***) face-processing network. Each graph represents the mean β weight (with 95% confidence intervals) for female and male faces generated from a ROI-based GLM in the independently and individually defined face-related ROIs plotted as a function of participant sex. There were no sex differences in the magnitude of the response to female faces in any ROI.

To leverage the power of the full sample, we also investigated group differences in the magnitude and the size of category-selective activation within each ROI using separate two-tailed independent-samples *t*-tests. Consistent with the repeated-measures analyses, these *t*-tests revealed no group differences in the magnitude of face-, object-, or place-related activation in any ROI (Table 4). Similarly, there were no group differences in the extent of activation for any of the face-, object-, or place-related ROIs. We conducted a follow-up analysis on the extent of activation by excluding the individuals who had no super-threshold activation (i.e., no 0). The results did not change. There were no sex differences in the size of the functional activation in any ROI.

**Table 4.** Summary of results from series of independent-samples *t* tests comparing female and male participants on the size and magnitude of category-selective activation during the visual stimulation experiment and the OGB in the magnitude of activation elicited during the identity recognition experiment within each ROI

	Visual stimulation task						Scanner recognition task		
	Female > male						OGB		
	**Size**			**Magnitude**			**Magnitude**		
Category	*t*	df	*p*	*t*	df	*p*	*t*	df	*p*
Faces									
Core regions									
R p-Fus (FFA2)	-0.008	28	0.993	0.809	16	0.430	-0.283	15	0.781
L p-Fus (FFA2)	-1.612	28	0.118	-0.319	16	0.754	0.370	15	0.717
R m-Fus (FFA1)	-1.320	28	0.198	-1.004	28	0.324	0.366	28	0.717
L m-Fus (FFA1)	-1.005	28	0.324	-0.540	28	0.593	-0.603	27	0.552
R pFG (IOG)	-0.618	28	0.542	0.988	27	0.332	-1.005	26	0.324
L pFG (IOG)	-0.471	28	0.641	-0.133	26	0.896	-0.572	25	0.573
R OFA	-0.186	28	0.853	-0.261	26	0.796	0.186	27	0.853
L OFA	-0.035	28	0.972	-1.189	25	0.246	1.620	26	0.117
R STS	-0.338	28	0.738	-0.598	28	0.554	1.640	27	0.113
L STS	1.026	28	0.314	0.646	26	0.524	2.476	27	0.020
Extended regions									
vmPFC	-0.665	28	0.512	-0.355	23	0.726	2.711	22	0.013
PCC	0.816	28	0.421	0.298	21	0.769	2.725	20	0.013
R Amyg	-0.908	28	0.371	0.933	20	0.362	0.319	19	0.753
L Amyg	-1.004	28	0.324	1.350	18	0.193	0.754	17	0.461
R ATL	-0.297	28	0.769	0.468	23	0.644	0.483	22	0.634
L ATL	0.037	28	0.971	0.561	14	0.583	0.285	13	0.781
Places									
R PPA	0.615	28	0.544	0.160	25	0.874	-1.816	24	0.082
L PPA	1.322	28	0.197	0.028	21	0.978	-1.618	21	0.121
Objects									
R LOC	2.424	28	0.022	-1.302	26	0.204	-3.362	25	0.002
L LOC	0.773	28	0.446	-0.365	27	0.718	-1.853	26	0.075

For the visual stimulation task, face-selective activation defined for face ROIs, object-selective activation defined for object ROIs and place-selective activation defined for place ROIs. The OGB was defined relative to each participant’s sex as a difference score in the magnitude of activation in response to the male and female face recognition blocks (e.g., for a female participant female > male blocks). The *t* tests evaluated a difference in magnitude of this relative OGB between the two sexes. Bolded *p* values surpassed the FWE correction of *p* < 0.004. All *t* tests were independent-samples two-tailed tests.

These results demonstrate that during passive viewing of dynamic faces, objects, and places, biological sex does not influence the magnitude or extent of functional neural responses to faces, objects, or places. These results indicate that the basic topography of the ventral visual pathway does not appear to be influenced by biological sex.

### Biological sex as a factor influencing the neural basis of face recognition

Participants also performed a separate Identity Recognition task in the scanner that was designed to be computationally similar to block 4 of the CFMT+. We submitted the raw accuracy scores to a repeated-measures ANOVA with the between-subjects factor of participant sex and the within-subjects factor of stimulus sex. As in the behavioral tasks outside the scanner, there was a main effect of stimulus sex, *F*_(1,28)_ = 4.38, *p* < 0.05. Both male and female participants were more successful recognizing the male (M = 83.2, SD = 15.8) compared with female (M = 77.0, SD = 20.7) exemplar face. As a result, it was impossible to acquire an unbiased estimate of the OGB. As with the behavioral data acquired outside the scanner, we standardized the raw accuracy scores to address this difference in difficulty so that we could acquire an unbiased estimate of the OGB for both men and women. When we analyzed the *z*-transformed mean accuracy scores, there were no main effects of stimulus sex, *F*_(1,28)_ = 0.00, *p* = 1.0, or group, *F*_(1,28)_ = 0.15, *p* = 0.70, on behavioral performance. However, there was a stimulus sex × participant sex interaction, *F*_(1,28)_ = 5.20, *p* = 0.03, η^2^ = 0.16. To understand this interaction, we estimated the magnitude of the potential OGB in male and female participants separately using paired-samples one-tailed *t*-tests comparing the *z*-scored recognition accuracy for male and female faces in the sex-specific direction of an OGB (e.g., for female participants female - male recognition accuracy). Neither female, *t*_(14)_ = 1.74, *p* = 0.052, nor male, *t*_(14)_ = 1.5, *p* = 0.08, participants exhibited a significant pattern of OGB in their recognition behavior during the scanner recognition task. Given the statistical trend for this OGB effect in both groups of participants, we compared the magnitude of the OGB in this task to that observed in the CFMTs. To do so, we computed sex-specific OGB difference scores from the raw accuracy data from each set of tasks (e.g., for male participants male face recognition-female face recognition) and *z*-scored these data. We submitted the *z*-scores to separate repeated-measures ANOVAs including the within-subjects factor of task for each group. These analyses revealed no main effect of task for either the men, *F*_(1,14)_ = 0.15, *p* = 0.71, or women, *F*_(1,14)_ = 1.54, *p* = 0.24, indicating that performance in the scanner task for both groups was not different from the performance observed during CFMTs. In other words, there was consistency across both male and female participants and across tasks for the lack of an OGB.

To evaluate whether biological sex is related to the magnitude of activation during this face recognition task, we investigated the main effects and interactions of biological sex with stimulus sex and hemisphere separately in pairs of bilateral individually defined ROIs. The full set of results is reported in Table 5. The only main effect of stimulus sex was in the LOC in which female faces elicited stronger activation in both male and female participants (*p* = 0.004). Importantly, there were neither main effects of group nor interactions between group and stimulus sex nor hemisphere in any ROI (*p* > 0.004; Fig. 5*A*,*B*). This reveals that there was no influence of biological sex on the magnitude of neural activation during face recognition. Also, there was no pattern of OGB in the neural responses for either men or women. Specifically, neither males nor females exhibited higher neural responses to their respective same-sex faces during recognition in the scanner (Fig. 5*A*,*B*).

**Table 5.** Summary of results from repeated-measures ANOVAs on the magnitude of face activation during the identity recognition experiment in each pair of bilateral ROIs

Category		df	*F*	*p*
Faces				
Core regions				
p-Fus (FFA2)	*Group*	1, 12	3.823	0.074
	*Hem*	1, 12	0.309	0.588
	*Stim Sex*	1, 12	0.484	0.500
	*Stim Sex* × *Hem*	1, 12	0.549	0.473
	*Stim Sex* × *Group*	1, 12	0.097	0.760
	*Group* × *Hem*	1, 12	0.739	0.407
	*Group* × *Hem* × *Stim Sex*	1, 12	0.006	0.942
m-Fus (FFA1)	*Group*	1, 28	0.145	0.706
	*Hem*	1, 28	6.007	0.021
	*Stim Sex*	1, 28	0.956	0.337
	*Stim Sex* × *Hem*	1, 28	0.258	0.615
	*Stim Sex* × *Group*	1, 28	0.210	0.651
	*Group* × *Hem*	1, 28	0.080	0.780
	*Group* × *Hem* × *Stim Sex*	1, 28	0.113	0.739
pFG (IOG)	*Group*	1, 25	0.339	0.566
	*Hem*	1, 25	0.014	0.906
	*Stim Sex*	1, 25	0.736	0.399
	*Stim Sex* × *Hem*	1, 25	0.149	0.703
	*Stim Sex* × *Group*	1, 25	0.029	0.866
	*Group* × *Hem*	1, 25	1.385	0.250
	*Group* × *Hem* × *Stim Sex*	1, 25	0.215	0.647
OFA	*Group*	1, 23	1.628	0.215
	***Hem***	**1, 23**	**12.688**	**0.002**
	*Stim Sex*	1, 23	0.245	0.626
	*Stim Sex* × *Hem*	1, 23	2.902	0.102
	*Stim Sex* × *Group*	1, 23	0.119	0.733
	*Group* × *Hem*	1, 23	1.016	0.324
	*Group* × *Hem* × *Stim Sex*	1, 23	0.088	0.770
STS	*Group*	1, 26	0.541	0.468
	*Hem*	1, 26	0.421	0.522
	*Stim Sex*	1, 26	3.953	0.057
	*Stim Sex* × *Hem*	1, 26	1.757	0.197
	*Stim Sex* × *Group*	1, 26	0.053	0.820
	*Group* × *Hem*	1, 26	0.000	0.994
	*Group* × *Hem* × *Stim Sex*	1, 26	0.052	0.822
Extended regions				
Amyg	*Group*	1, 17	1.607	0.222
	*Hem*	1, 17	0.002	0.966
	*Stim Sex*	1, 17	0.560	0.464
	*Stim Sex* × *Hem*	1, 17	0.407	0.532
	*Stim Sex* × *Group*	1, 17	1.661	0.215
	*Group* × *Hem*	1, 17	0.148	0.705
	*Group* × *Hem* × *Stim Sex*	1, 17	3.909	0.064
ATL	*Group*	1, 13	0.005	0.945
	*Hem*	1, 13	1.116	0.310
	*Stim Sex*	1, 13	1.141	0.305
	*Stim Sex* × *Hem*	1, 13	0.930	0.352
	*Stim Sex* × *Group*	1, 13	0.039	0.847
	*Group* × *Hem*	1, 13	0.041	0.844
	*Group* × *Hem* × *Stim Sex*	1, 13	0.935	0.351

Places				
PPA	*Group*	1, 21	0.277	0.604
	*Hem*	1, 21	0.600	0.447
	*Stim Sex*	1, 21	4.797	0.040
	*Stim Sex* × *Hem*	1, 21	0.779	0.387
	*Stim Sex* × *Group*	1, 21	0.000	0.999
	*Group* × *Hem*	1, 21	1.287	0.269
	*Group* × *Hem* × *Stim Sex*	1, 21	3.653	0.070
Objects				
LOC	*Group*	1, 26	0.099	0.755
	*Hem*	1, 26	0.098	0.757
	***Stim Sex***	**1, 26**	**9.922**	**0.004**
	*Stim Sex* × *Hem*	1, 26	1.765	0.196
	*Stim Sex* × *Group*	1, 26	2.240	0.146
	*Group* × *Hem*	1, 26	0.081	0.778
	*Group* × *Hem* × *Stim Sex*	1, 26	0.882	0.356

Face-selective activation defined for face ROIs, object-selective activation defined for object ROIs and place-selective activation defined for place ROIs. Bolded *p* values surpassed the FWE correction of *p* < 0.004.

To leverage the power of the full sample, we also investigated group differences in the magnitude of the OGB within each ROI using separate two-tailed independent-samples *t* tests (Table 4). There were no sex differences in the magnitude of the OGB in any ROI. In the right LOC, both groups exhibited a higher magnitude responses for female compared with male faces, which is consistent with the repeated-measures analyses.

## Discussion

We investigated the influence of biological sex on the behavioral and neural basis of recognition for male and female faces and objects in typically developing, healthy, young adults. This included evaluating the topography of ventral stream functional organization for faces, objects, and places as well as neural activation within the face-processing network specifically during face recognition.

### The influence of biological sex on recognition behavior

To investigate the influence of biological sex on the specificity of recognition behavior, we used the M-CFMT+ and CCMT, and we created the F-CFMT+. Reliability of performance on the F-CFMT+ was similar to the M-CFMT+ in our sample and to that of the original M-CFMT short form (Duchaine and Nakayama, 2006). We observed no sex differences in performance on either the M-CFMT+ or the F-CFMT+ in either the large sample or the subsample of scanned participants. Both male and female participants performed more accurately on the F-CFMT+ than on the M-CFMT+. To control for task demands, we *z*-scored data from all three recognition tasks and evaluated sex differences on the M-CFMT+ and F-CFMT+ using the CCMT as a covariate to control for the general task demands (memory, speeded recognition). These analyses revealed no effects of biological sex on recognition of either male or female faces.

Therefore, the differences in performance on these two face recognition tasks, as reflected in the raw accuracy scores, are likely due to task-related factors. To evaluate this possibility, we compared the accuracy of responses to each of the target faces in both versions of the CFMT. We found that four of the six female target faces were easier to distinguish from distractors compared with the comparable target male faces. Only two of the female target faces were comparably matched in difficulty to the parallel target male faces. One interpretation of these findings is that we inadvertently picked female target faces that are relatively more distinctive compared with the female distractor faces than are the comparable male target faces among their male distractor faces. We are pursuing this hypothesis in future versions of the task. Critically, the recognition behavior of both male and female participants was similarly impacted by task-related differences; participant sex did not interact with the task parameters.

A second goal was to investigate the presence of an OGB in both groups of participants. Specifically, we evaluated whether female participants exhibit superior recognition for female compared with male faces and/or male participants exhibit superior recognition for male compared with female faces. The analyses with the *z*-scored data revealed that neither group exhibited an OGB in their pattern of recognition behavior in the CFMTs or in the scanner recognition task.

In contrast to the previous, albeit conflicted, literature we did not replicate studies reporting an OGB in women (Herlitz and Lovén, 2013). Importantly, the task effect whereby both male and female participants exhibited better performance recognizing female compared with male faces has been reported in other studies using similar measures (Lovén et al., 2011). In some of these studies, the authors interpreted their findings to reflect a selective OGB in the females, but not males, instead of a main effect of task. We suggest that it is critical to control all task-related factors and to match tasks and stimuli on difficulty a priori before any claims can be evaluated regarding the potential influence of biological sex. Our findings indicate that the stimuli and task parameters likely influence the conditions under which an OGB may be observed and interpreted. As a result, we suggest that there needs to be more theoretical consideration about the nature and mechanisms of the OGB in face recognition.

With respect to object recognition, males were more accurate on the CCMT than were females, which is consistent with previous findings (Dennett et al., 2011). However, the sex difference in object recognition was not present in the scanned subsample of participants, indicating that it may not be particularly robust.

### No influence of biological sex on the neural topography of the ventral visual pathway

We addressed whether there are potential sex differences in the topography of the category-specific functional organization within the ventral visual pathway. We measured the magnitude and extent of individually defined face-, object-, and place-related regions bilaterally. We found no sex differences in any of these measures of neural function in any of the 20 ROIs or in the whole-brain group level comparisons. These findings reveal, for the first time, that men and women have similar category-selective topographic organization in the ventral visual pathway. This means that both core and extended face-related regions, as well as object- and place-related regions, are comparable in the spatial organization and magnitude of category-selective activation across both hemispheres in men and women.

These findings do not replicate reported sex differences in the magnitude of face-related activation in the fusiform and inferior occipital gyri (Lovén et al., 2014) and amygdala (Fischer et al., 2004; Killgore et al., 2001) in adults, which has also been reported in the amygdala (Schneider et al., 2011) and bilateral FFA and LOC (Tahmasebi et al., 2012) in adolescents. Importantly, although each of these studies reported sex differences in the magnitude of activation during face processing, there is little convergence across the studies regarding the directionality of the sex difference within or between any of the regions. Also, none of the existing studies defined the ROIs on an individual subject basis with rigorous correction for false positive activation. As a result, we suggest that the convergence of findings in our study across the two measures of neural activation (magnitude, extent) for three kinds of category-specific activation (faces, places, objects) in all 20 ROIs, which were corrected at the whole-brain level for false positive activation, are the most methodologically rigorious to date. Our findings indicate that biological sex does not influence the functional organization of category-selective activation within the ventral visual pathway, for faces, objects, or places.

### No influence of biological sex on the neural basis of face recognition

Second, to investigate the influence of biological sex on neural activation during face recognition, we trained participants to recognize a single target male and female face before scanning. During scanning, participants recognized novel exemplars of these two target faces in separate blocks of male and female distractor faces. We extracted β weights for the male and female faces from the independently defined ROIs to evaluate whether there are sex differences in the magnitude of the responses during recognition of either sex face and/or evidence of an OGB in the magnitude of the neural responses. We did not observe sex differences in the magnitude of neural responses during recognition of either male or female faces in any ROI. Also, there was no OGB in the neural responses of either male or female participants.

These findings are inconsistent with two previous reports of an OGB in the neural responses of female participants in the bilateral fusiform and inferior occipital gyri (Lovén et al., 2014) and amygdala (Armony and Sergerie, 2007), which may be explained by methodological differences between these studies and the current study. For example, the ROIs for analysis of the OGB in the previous studies were not defined independently from the contrast used to select the voxels, which distorts (usually by inflating) the magnitude of the dependent effect under investigation (Kriegeskorte et al., 2010). Also, the analyses within the ROIs were not corrected for false positive activation, which can also lead to spurious results (Bennett et al., 2009). In the Lovén et al. (2014) study, males also exhibited higher magnitude responses to female compared with male faces in the same ROIs. This suggests that the pattern of results in the females reflected neural responses to differences in the stimuli that were shared by the male participants, not a sex-specific OGB.

Finally, previous work reporting sex differences in neural activation underlying face processing has neglected to screen participants for a history of concussions and subclinical behaviors indicative of a potential psychiatric diagnosis. Concussions often cause widespread visual dysfunction (Barnett and Singman, 2015) and multiple aspects of face perception are disrupted in every social-emotional disorder (e.g., anxiety, depression, bipolar, schizophrenia, autism). We suggest that some of the effects that have been reported previously as sex differences may instead reflect differences in health histories.

### Limitations and future directions

It is important to note that while our study employed a sample size (*N* = 30) larger than any previous fMRI study investigating the influence of sex on face recognition (Fischer et al., 2004; Lovén et al., 2014), we acknowledge that expectations are changing regarding sample sizes in fMRI studies comparing groups. This speaks to concerns about whether our experimental design was underpowered to detect a true sex difference in the scanner tasks (i.e., Type II error). It is important to remember that improving sensitivity to detect an effect is accomplished in two ways: by (1) increasing the sample size and/or (2) increasing sensitivity of the measures (e.g., decreasing error/measurement noise). Recall that we employed a within-subjects design and scanned each of our participants in two separate tasks (visual stimulation, face recognition), using two dependent measures of neural activation (magnitude, extent), in 16 independently, a priori defined face-related regions and four control regions using state-of-the-art correction procedures. In addition, in our analyses of the neuroimaging data, we followed the most recent recommendations about how to improve statistical power when sample sizes are lower (Poldrack et al., 2017). These include collecting much larger data sets from each individual participant and presenting the findings at the individual level rather than at the group level, using a more liberal statistical thresholding procedure like the FDR, and restricting the search space for group comparisons using an independent and a priori voxel selection (ROI) procedure (Poldrack et al., 2017). As a result, despite the relatively small sample size by newer field standards, we have conducted the most methodologically rigorous test of the influence of sex on the behavioral and neural basis of face recognition in men and women to date. Moving forward, we encourage researchers to use a similar methodological approach with a larger sample size in future work. Importantly, we also encourage researchers to pre-register the methodological and data analysis plans for studies investigating the influence of biological sex on both the behavioral and neural basis of face recognition abilities given the discrepancies in the literature.

There are several aspects about the experimental design and analyses that we would like to build on going forward. Specifically, the recognition task in the scanner required participants to recognize multiple exemplars of one male and one female identity, which represents a true test of identity recognition accuracy. In future versions of the task, we would like to employ multiple identities from each sex to test more broad face recognition abilities and unconfound face sex with face identity in this task. In addition, although the current analyses indicate that activation within the nodes of the face-processing network do not differ as a function of sex during visual stimulation or during face recognition, it may still be the case that the patterns of functional connections between these nodes may vary as a function of sex during these tasks, which we will address in future research. Finally, we will continue to improve the F-CFMT+ so that it is matched in difficultly with the M-CFMT+, which will enable unbiased estimates of the OGB to be measured in the raw scores for both men and women.

### Conclusions

Our findings suggest that face recognition behavior, including the OGB, is not inherently sexually dimorphic. Face recognition is an essential skill for navigating human social interactions, which is reflected equally in the behavior and neural architecture of men and women. Instead, we predict that there may be particular contexts in which sex differences in face recognition and an OGB can be dynamically elicited (Motta-Mena et al., 2016). Determining such contexts is critical for future investigations.

## Synthesis

Reviewing Editor: Bradley Postle, University of Wisconsin Decisions are customarily a result of the Reviewing Editor and the peer reviewers coming together and discussing their recommendations until a consensus is reached. When revisions are invited, a fact-based synthesis statement explaining their decision and outlining what is needed to prepare a revision will be listed below. Note: If this manuscript was transferred from JNeurosci and a decision was made to accept the manuscript without peer review, a brief statement to this effect will instead be what is listed below.

After reading authors' response to the second round of reviews, it's my assessment that the authors have satisfactorily addressed the reviewers' concerns. Importantly, in cases where the authors convincingly rebutted concerns raised by the reviewers, the revised manuscript now explicitly articulates each of these as a possible concern, then explains why this concern does not apply to this study.
